# “Beige” Cross Talk Between the Immune System and Metabolism

**DOI:** 10.3389/fendo.2019.00369

**Published:** 2019-06-18

**Authors:** Krisztina Banfai, David Ernszt, Attila Pap, Peter Bai, Kitti Garai, Djeda Belharazem, Judit E. Pongracz, Krisztian Kvell

**Affiliations:** ^1^Department of Pharmaceutical Biotechnology, Faculty of Pharmacy, University of Pécs, Pécs, Hungary; ^2^Szentagothai Research Center, University of Pécs, Pécs, Hungary; ^3^Department of Physiology, Medical School, University of Pécs, Pécs, Hungary; ^4^Department of Biochemistry and Molecular Biology, Faculty of Medicine, University of Debrecen, Debrecen, Hungary; ^5^Medical Chemistry, Faculty of Medicine, University of Debrecen, Debrecen, Hungary; ^6^MTA-DE Cell Biology and Signaling Research Group, Debrecen, Hungary; ^7^MTA-DE Lendulet Laboratory of Cellular Metabolism, Debrecen, Hungary; ^8^Research Center for Molecular Medicine, University of Debrecen, Debrecen, Hungary; ^9^Department of Pathology, University Hospital of Mannheim, Mannheim, Germany

**Keywords:** thymus senescence, beige adipose tissue, TBX-1, UCP-1, PPARgamma

## Abstract

With thymic senescence the epithelial network shrinks to be replaced by adipose tissue. Transcription factor TBX-1 controls thymus organogenesis, however, the same TBX-1 has also been reported to orchestrate beige adipose tissue development. Given these different roles of TBX-1, we have assessed if thymic TBX-1 expression persists and demonstrates this dualism during adulthood. We have also checked whether thymic adipose involution could yield beige adipose tissue. We have used adult mouse and human thymus tissue from various ages to evaluate the kinetics of TBX-1 expression, as well as mouse (TEP1) and human (1889c) thymic epithelial cells (TECs) for our studies. Electron micrographs show multi-locular lipid deposits typical of beige adipose cells. Histology staining shows the accumulation of neutral lipid deposits. qPCR measurements show persistent and/or elevating levels of beige-specific and beige-indicative markers (TBX-1, EAR-2, UCP-1, PPAR-gamma). We have performed miRNome profiling using qPCR-based QuantStudio platform and amplification-free NanoString platform. We have observed characteristic alterations, including increased miR21 level (promoting adipose tissue development) and decreased miR34a level (bias toward beige adipose tissue differentiation). Finally, using the Seahorse metabolic platform we have recorded a metabolic profile (OCR/ECAR ratio) indicative of beige adipose tissue. In summary, our results support that thymic adipose tissue emerging with senescence is *bona fide* beige adipose tissue. Our data show how the borders blur between a key immune tissue (the thymus) and a key metabolic tissue (beige adipose tissue) with senescence. Our work contributes to the understanding of cross talk between the immune system and metabolism.

## Introduction

In human the degenerative process of thymic adipose involution is already detectable in childhood and accelerates with puberty due to hormonal (sex-steroid) induction (1–3). The process shows identical kinetics in mouse. Also, we have developed a model whereby TECs are treated by a steroid (using Dx or dexamethasone) thus both *in vivo* and *in vitro* model systems are readily available (4) As for all adipose tissues subtypes, thymic adipose involution is orchestrated by transcription factor PPARgamma (5–7). It is estimated that by the age of 50 years in human (approx. 12 months in mouse), the thymus loses approx. Ninety percent of its function: naïve T-cell production (8, 9). The consequences of impaired thymus function are profound: elevated incidence of infections, cancer and autoimmune disorders observed at senior ages (10, 11). This poses a significant burden on health-care and health-insurance systems, while simultaneously lowering the quality of life in the elderly.

Transcription factor TBX-1 is a key molecular player in the formation of the third pharyngeal pouch involved in thymus organogenesis during embryonic development (12). Human patients with 22q11.2DS impairing TBX-1 often have thymus hypoplasia or aplasia. In accordance, Tbx-1^null^ mice develop severe pathologies in tissues derived from the third pharyngeal pouch, including hypoplasia of the thymus (13, 14). In these cases, impaired thymus organogenesis leads to deficient thymocyte development, naive T-cell production, and immune functions (15). However, recently it has also been reported that the role of TBX-1 in thymus organogenesis is more complex. Ectopic expression of TBX-1 may suppress transcription factor FoxN1, the mastermind of thymic epithelial identity (16). The issue was investigated in the embryonic setting, but the potential role of persistent TBX-1 expression during adulthood has not been addressed.

TBX-1 has another pivotal role in the development and function of a recently described subtype of adipose tissue: beige adipose tissue (17–20). White adipose tissue stores energy, brown adipose tissue generates heat (via NST or non-shivering thermogenesis), while beige adipocytes act as intermediates. Beige adipocytes respond to adrenergic stimuli by thermogenesis (21). TBX-1 is considered as a beige-specific marker, but other beige-indicative markers have also been described. Mitochondrial uncoupling proteins (mostly UCP-1) have been reported to be expressed by brown / beige adipose tissue. EAR2 (also known as Nr2f6) was reported to efficiently promote adipose tissue development with beige bias, while CD137 (also known as Tnfrsf9) is an acknowledged beige adipocyte surface marker (22).

The adult thymus expresses TBX-1 and UCP-1 in the stromal compartment, both known to promote beige adipose tissue development. Yet to date thymic adipose tissue that develops with age has not been accurately positioned on this white-beige-brown continuum of adipose tissue subtypes, despite recent cellular analysis from an adipocyte perspective (23–26). For this reason, we have characterized senescence-related thymic adipose tissue using molecular, cellular and histological markers, at structural and ultra-structural levels, using both mouse and human samples. Additionally, we have also performed metabolic profiling and complete miRNome analysis using both PCR-based and amplification-free platforms.

## Methods

### Cell Cultures

For *in vitro* experiments primary-derived (BALB/c) thymic epithelial cells were used (TEP1) as reported previously (cell source: Prof. G. Anderson, University of Birmingham, UK) (27). Briefly, the cells were cultured in DMEM (Dulbecco's Modified Eagle's medium Lonza) supplemented with 10% FCS, penicillin, streptomycin and β-mercapto-ethanol. Human thymus-derived 1889c thymic carcinoma cells were cultured in RPMI 1640 (Roswell Park Memorial Institute medium, Lonza) containing 10% FCS, penicillin, streptomycin, L-Glutamine and Hepes (28, 29). Adipose differentiation of TEP1 and 1889c cells was induced by steroid treatment. Briefly, experiments differentiation was induced by dexamethasone alone (Dx) as added to complete DMEM and RPMI medium. Cells were treated with Dx at a final concentration of 1 μM for 1 week.

### Animal Samples

Thymus lobes were used from C57BL/6J mice at 1, 6, 8, 12, 14, 18, and 21 months of age. Mice were housed under minimal disease (MD) conditions. Animal rooms were ventilated 15 times/h with filtered air, mice received autoclaved pellet diet (Altromin VRF1) and tap water *ad libitum*. The cages contained sterilized bedding. Room lighting was automated with 12 h light and 12 h dark periods. Room temperature was 21 ± 2°C, relative humidity was between 30 and 60%. Mice were kept in the Laboratory Animal Core Facility of the University. Experimental procedures were carried out according to the “1988/XXVIII act of the Hungarian Parliament on Animal Protection (243/1988)” which complies with recommendations of the Helsinki Declaration. All animal experiments were performed with the consent of the Ethics Committee on Animal Research of the University (ref. no.: #BA02/2000-46/2016).

### Enrichment of Primary Cells

Mouse thymic epithelial cells were isolated by MACS cell separation. Briefly, mouse thymic lobes (1 month-old or 12 month-old) were digested with type F collagenase from C. hystolyticum (3mg/ml, Sigma-Aldrich) for 2 h, with stirring in every 20 min, then washed with DMEM. Cell suspensions were then labeled with anti-EpCAM1 antibody (1:100, rat monoclonal antibody clone: G8.8) and washed with MACS-buffer (2% FCS, 1mM EDTA in PBS) followed by incubation with Dynabeads sheep anti-rat IgG-coated beads (Invitrogen) The EpCAM+-cells were separated with EasySep column-free cell isolation platform (Stemcell Technologies) according to the manufacturer's instructions. Isolated cells were used for total RNA isolation and subsequent qPCR analysis.

### Human Thymus Samples

Formalin-fixed, paraffin-embedded (FFPE) human thymus samples from 18, 23, 42, 44, and 58 years of age were provided by the Department of Pathology, Faculty of Medicine, University of Pecs, Hungary. Experiments involving human samples were performed with the consent of the Regional and Local Ethics Committee of Clinical Center of the University (ref. no.: 6069/2016) according to their guidelines. All subjects gave written informed consent in accordance with the Declaration of Helsinki.

### Transmission Electron Microscopy

Cells were harvested and pelleted then fixed with PBS containing 2.5% glutaraldehyde overnight at 4°C. Following fixation, pellets were mixed in 3% porcine gelatin (Sigma-Aldrich). Hardened small blocks of approximately 1 mm^3^ were cut. Blocks were post-fixed in 1% osmium-tetroxide in PBS for 1 h at 4°C and dehydrated with increasing concentration of ethanol. Uranyl-acetate (1%) was added in 70% ethanol to increase contrast. After complete dehydration in ascending ethanol series, blocks were transferred to propylene-oxide twice for 4 min. Then blocks were immersed in the mixture of propylene-oxide and Durcupan resin (Sigma-Aldrich) for 30 min. Later blocks were placed into Durcupan-containing tin-foil boats overnight, and embedded into gelatin capsule filled with Durcupan resin (Sigma-Aldrich). Following polymerization and hardening of the resin at 56 °C for 72 h, semi thin sections were cut with Leica Ultracut ultramicrotom, mounted on glass slides, stained with toluidine-blue and examined with Olympus BX50 light microscope. Then serial ultra-thin sections were cut by ultramicrotom, and mounted on mesh grids. Ultra-thin sections were contrasted by uranyl-acetate and lead-citrate, and examined using JEOL 1200EX-II electron microscope.

### Immune-Histochemistry

Human thymus lobes were fixed in paraformaldehyde (4% PFA in PBS) then paraffin embedded. 5μm thick sections were stained with immunohistochemistry method as described earlier (30). First, slides were rinsed in heated xylene then washed with a descending series of alcohol. After deparaffinization slides were rehydrated and antigen retrieval was performed in Target Retrieval Solution (pH 6 DAKO) at 97°C for 20–30 min. Following wash in dH_2_O and endogenous peroxidase activity was blocked with 3% H_2_O_2_ in TBS (pH 7.4) for 15 min. Then slides were washed with TBS containing Tween (0.05%, pH 7.4). Pre-blocking was carried out with 3% BSA in TBS for 20 min followed by overnight incubation with a-TBX-1 (1:100, rabbit polyclonal antibody, Sigma-Aldrich) primary antibody at 4°C. After the incubation slides were washed with TBS then incubated with peroxidase conjugated secondary antibody (1:100, Polyclonal Goat Anti-Rabbit IgG, DAKO) for 90 min. Labeling was visualized with liquid DAB Substrate Chromogen System (DAKO). Hematoxylin served for nuclear counterstaining. Slides were mounted with Faramount Aqueous Mounting Medium (DAKO). Histological evaluation was performed with Panoramic MIDI digital slide scanner (3DHistech) and images were captured with CaseViewer. Image analysis was made with ImageJ / IHC toolbox.

### Immune-Fluorescent Staining

Immune-fluorescent staining was performed on 8μm cryostat thymus sections. Cytospin technique was used to spin TEP1 and 1889c cells onto glass slides (4). Slides were fixed in cold acetone, then dried and blocked using 5% BSA in PBS for 20 min before staining with fluorochrome conjugated or primary antibodies: a-EpCAM-FITC (1:100, clone: G8.8,), a-UCP-1 (1:100, rabbit polyclonal antibody, Abcam) a-TBX-1 (1:100, rabbit polyclonal antibody, Sigma-Aldrich), a-PPAR-gamma (1:100, rabbit monoclonal antibody, Cell Signaling Technology). For secondary antibody Alexa-555 conjugated a-rabbit goat IgG (1:200, Life Technologies) was used. Fluorescent lipid staining was performed on paraformaldehyde (4%) fixed TEP1 and 1889c cytospin slides with LipidTOX Red dye (1:200, Invitrogen). For nuclear counterstain DAPI (Life Technologies) was used. Sections were imaged using a Nikon Eclipse Ti-U microscope equipped with a CCD camera (Andor Zyla 5.5) and NIS-Elements software.

### Metabolic Profiling

The use of TEP1 cells for Seahorse metabolic profiling was started by pilot experiments for optimal starting cell number, duration of differentiation, differentiation medium etc. Accordingly, 15,000 cells/well were cultured for 9 days using standard MDI differentiation protocol (31). This was followed by the evaluation of their metabolic profile using the Seahorse XF 96 platform (Seahorse Bioscience). Cells were plated into Seahorse cell plates at confluence and were left to attach overnight. The next day, cells were subject to oxymetry measurement. After recording baseline oxygen consumption cells were treated with butyril-cAMP (500 μM), oligomycin (2 μM), and antimycin (10 μM). Antimycin-resistant oxygen consumption was considered as background and was subtracted from all values. Baseline oxygen consumption, membrane leak (OCR, after oligomycin treatment) was calculated. Glycolysis was assessed through the extracellular acidification value (ECAR, before oligomycin treatment) and the ECAR/OCR values were calculated. Negative values were omitted in calculations.

### RNA Isolation, cDNA Preparation, qRT-PCR, TaqMan Array

Total RNA of enriched thymic epithelial cells, TEP1 and 1889c cells was isolated with the NucleoSpin RNAII kit (Macherey-Nagel). cDNA was prepared using the High Capacity cDNA Reverse Transcription kit (Applied Biosystems). For qPCR analysis the StepOnePlus (Applied Biosystems) platform was used with SensiFAST SYBR Hi-ROX Mix (Bioline) as well as PikoReal™ Real-Time PCR System (Thermo Fisher Scientific) using Luminaris Color HiGreen qPCR Master Mix (Thermo Fisher Scientific) (for primer list see Table 1). Gene expression was normalized to β-actin, GUSB and HPRT1 housekeeping genes. Reverse transcription of 1889c RNA samples for miRNA analysis was completed with Megaplex™ RT Primers specific to human Pool A (Cat. No.: 4399966) and Pool B (Cat. No.: 4444281). MiRNA profiling was performed on Applied Biosystems Quantstudio™ 12K Flex Real-Time PCR System platform using TaqMan™ Array Human MicroRNA A (Applied Biosystems, Cat. No.: 4398965) and B Card (Applied Biosystems, Cat. No.: 4444910) containing 6 housekeeping genes (RNU44, RNU48, ath-miR159a and 4 U6 snRNAs) and 377 human miRNAs. Additionally 600 ng of total RNA was mixed with TaqMan™ Fast Universal PCR Master Mix (2X), no AmpErase™ UNG (Applied Biosystems, Cat. No.: 4364103) for each array card. Gene expressions were analyzed using Expression Suite Software version 1.1.

**Table 1:** List of mouse and human primer sequences.

**Gene Name**	**Mouse primer sequence**	**Human primer sequence**
Actin-for	GGGAGGGTGAGGGACTTCC	GCGCGGCTACAGCTTCA
Actin-rev	TGGGCGCTTTTGACTCAGGA	CTTAATGTCACGCACGATTTCC
GUSB-for	AAATGGAGTGCGTGTTGGGT	GATGCTGTACCCCCAGGA
GUSB-rev	CGGTACCATTGCTGCTCGAA	GTCGGTTGTCAGAGAAGTCG
HPRT-for	TTGCTCGAGATGTCATGAAGGA	CTGGCGTCGTGATTAGTGAT
HPRT-rev	ATGTAATCCAGCAGGTCAGCA	ACATCTCGAGCAAGACGTTC
CD137-for	CGTGCAGAACTCCTGTGATAAC	CCTGAGCTACAAAGAGGACAC
CD137-rev	CTCCACCTATGCTGGAGAAGG	GTGCAGCGCAAGTGAAAC
Ear2-for	CCTGTACCCCAGAACTCCA	GCAAGCATTACGGTGTCTTC
Ear2-rev	CAGATGAGCAAAGGTGCAAA	GATCTGGCAGTCACGGTTG
PPARg-for	TGTCTCACAATGCCATCAGGT	GGTGGCCATCCGCATCT
PPARg-rev	TCTTTCCTGTCAAGATCGCCC	GCTTTTGGCATACTCTGTGATCTC
TBX1-for	GGCAGGCAGACGAATGTTC	CTACGACCACTATCTCGGGG
TBX1-rev	TTGTCATCTACGGGCACAAAG	TGGGGCAATAGTCGTAGGAG
UCP1-for	GGCCTCTACGACTCAGTCCA	ACAATCACCGCTGTGGTAAA
UCP1-rev	TAAGCCGGCTGAGATCTTGT	GTAGAGGCCGATCCTGAGAG

### NanoString System Assay

One hundred nanogram of total RNA was used to detect up to 800 miRNA targets with nCounter SPRINT Profiler (NanoString Technologies) using nCounter® Human v3 miRNA Expression Assay. Sample preparation was performed with nCounter® CodeSet (NanoString Technologies) following annealing, ligation and purification. Hybridization protocol was completed at 65°C and 12 h long according to the manufacturer's instructions. Quantified data was analyzed using nSolver™ Analysis Software version 4.0. Threshold count was determined using negative controls as background noise. Gene expression changes were visualized on heat map using GraphGad Prism version 7.04.

### Statistical Analysis

All experiments were performed at least on three occasions, representative experiments are shown. Measures were obtained in triplicates, data are presented as mean and ±SD as error bars. For statistical analysis GraphPad Prism software and SPSS Statistics version 22.0 was used. To evaluate the kinetics of TBX-1 expression with age in both mouse and human samples normality distribution was tested using Shapiro-Wilk test (*n* < 50). In case of human samples our data met the assumption of homogeneity of variances, so parametric one-way ANOVA with Tukey's honestly significant difference (HSD) *post hoc* test was used. To determine the significant differences of mouse samples non-parametric Kruskal-Wallis test was used. For further cases two-tailed student's *t*-test was applied. Significant differences are shown by asterisks (ns for *p* > 0.05, ^*^p ≤ 0.05, ^**^p ≤ 0.01, ^***^p ≤ 0.001).

## Results

### Aging and Steroid-Induced TECS Show Beige Adipocyte Markers

Thymic senescence is accompanied by the appearance of adipose tissue. Mediastinal location and local FGF21 production are characteristic to the thymus and both were reported to promote beige adipose tissue development (23–25). For this reason we searched for the up-regulation of beige adipocyte markers in the adult thymus tissue and its model system: steroid-induced TECs.

#### Aging Up-Regulates key Beige Adipocyte Marker in Human Thymus Tissue

**Figure 1:**
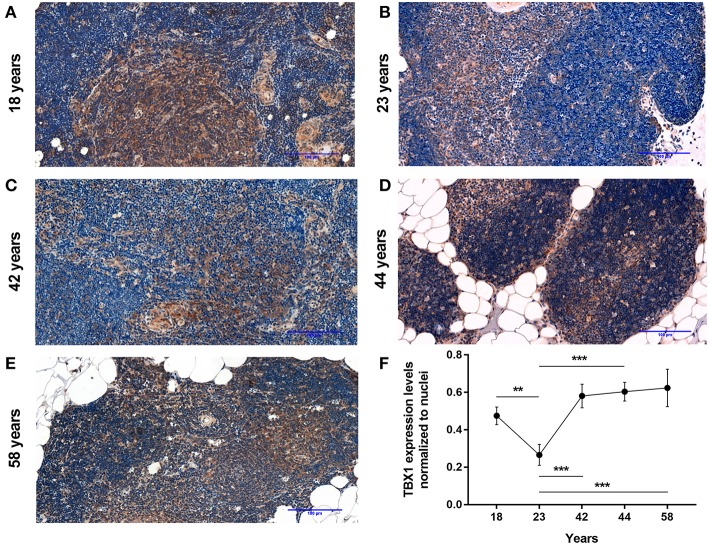
Kinetics of TBX-1 expression in the adult human thymus with age. Human thymic FFPE sections from different ages (18, 23, 42, 44, and 58 years) were evaluated by immune-histochemical staining **(A–E)**, respectively. Brown color reaction (DAB) shows TBX-1 expression along with hematoxylin nuclear counter-staining. Please note signs of adipose degeneration (vacuoles) at elevated ages. TBX-1 staining was normalized to nuclear counter-stain and is shown as relative value **(F)**. Please note that relative TBX-1 expression shows a transient decrease at young adult age (23 years of age). Significant differences are shown by asterisks (**p ≤ 0.01, ***p ≤ 0.001). Data were calculated from three slides and representative slide is shown. For exact numerical values and standard error of mean please refer to Supplementary Data Sheet.

Using a pilot set of human thymic FFPE samples of various adult ages (18, 23, 42, 44, and 58 years) we performed immune-histochemistry staining for beige adipose tissue-specific marker TBX-1 (Figures 1A–E). TBX-1 expression (enzyme reaction in brown) appears to persist throughout adulthood. Normalization to hematoxylin nuclear counterstain (in blue) shows that TBX-1 staining intensity transiently decreases at young adult age (23 years) to show rebound at later ages (Figure 1F). In other words: TBX-1 expression may present a bimodal nature with elevations at both young and adulthood ages and an in-between transient decrease during young adulthood. The histological appearance of adipocytes is observed from 44 years of age onwards in this series.

#### Further Beige Adipocyte Markers Are Also Up-Regulated in Steroid-Induced Human TECs

**Figure 2:**
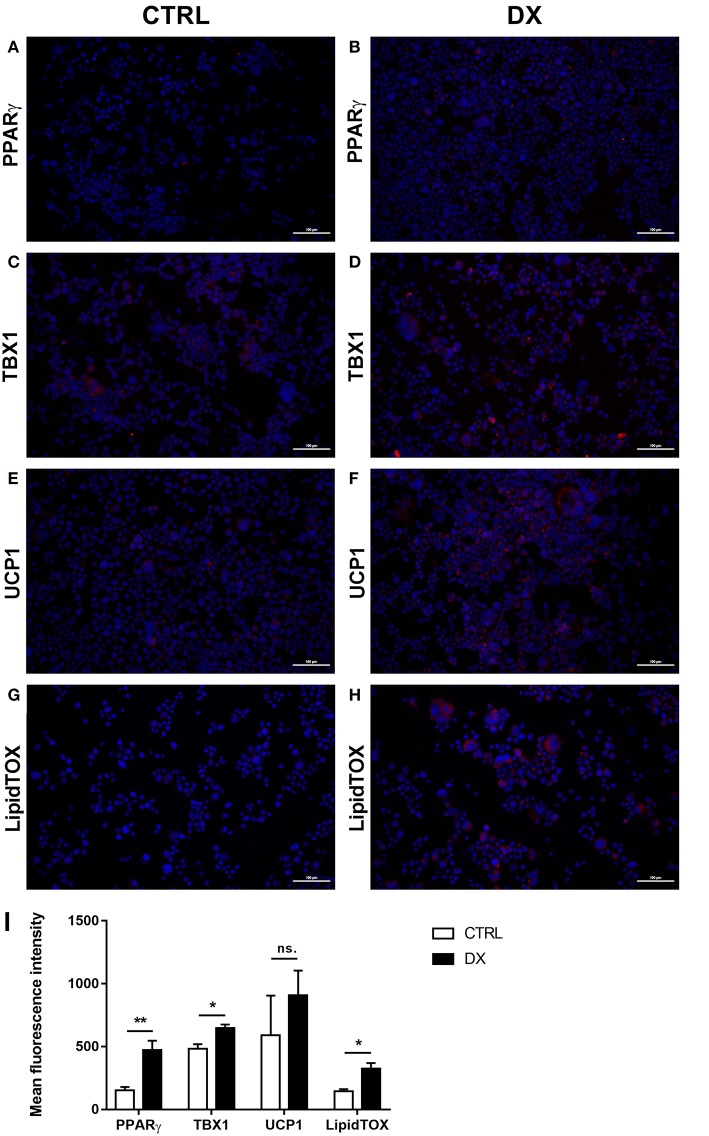
Beige adipocyte marker expression and lipid accumulation in steroid-induced human TECs. Cytospin slides of control (Ctrl) and steroid-induced (Dx) 1889c cells were stained by immune-fluorescence. Adipose tissue mastermind transcription factor PPARgamma **(A,B)**, beige-specific marker TBX-1 **(C,D)** and beige-indicative marker UCP-1 **(E,F)** was evaluated in red (Alexa555). Neutral lipid deposits were stained with LipidTOX Red dye **(G,H)**. DAPI staining was also applied as fluorescent nuclear counter stain in all cases. PPAR-gamma, TBX-1, UCP-1 and LipidTOX staining relative to DAPI staining is also shown by histograms **(I)**. PPAR-gamma and TBX-1 show significant increase, UCP-1 remains unchanged, while neutral lipid deposits show significant increase following Dx-induction. Significant differences are shown by asterisks (*p ≤ 0.05, **p ≤ 0.01). Data were calculated from six slides, representative slide is shown. For exact numerical values and standard error of mean please refer to Supplementary Data Sheet.

As reported previously molecular level events are similar in the aging thymus and steroid-induced TECs in the mouse setting (4, 6). Accordingly, Dx-treatment significantly up-regulated (*p* < 0.01) pan-adipocyte marker PPAR-gamma expression in the human 1889c TEC line (Figures 2A,B,I). We have evaluated 1889c human TECs for the expression of beige-specific and beige-indicative protein markers as well following Dx-treatment. Similar to human thymus sections above, 1889c cells showed persistent and increasing (*p* < 0.05) TBX-1 expression following Dx-treatment (Figures 2C,D,I). UCP-1 expression showed only indicative (not significant) increase upon Dx-treatment (Figures 2E,F,I). Lipid accumulation was also tested, using a fluorescent dye (LipidTox Red) specific for neutral lipid deposits. The staining showed that Dx-treatment triggers significant (*p* < 0.05) accumulation of neutral lipid deposits (Figures 2G–I) in harmony with our previous reports (4, 6).

#### Aging Up-Regulates key Beige Adipocyte Marker in Mouse Thymus Tissue

**Figure 3:**
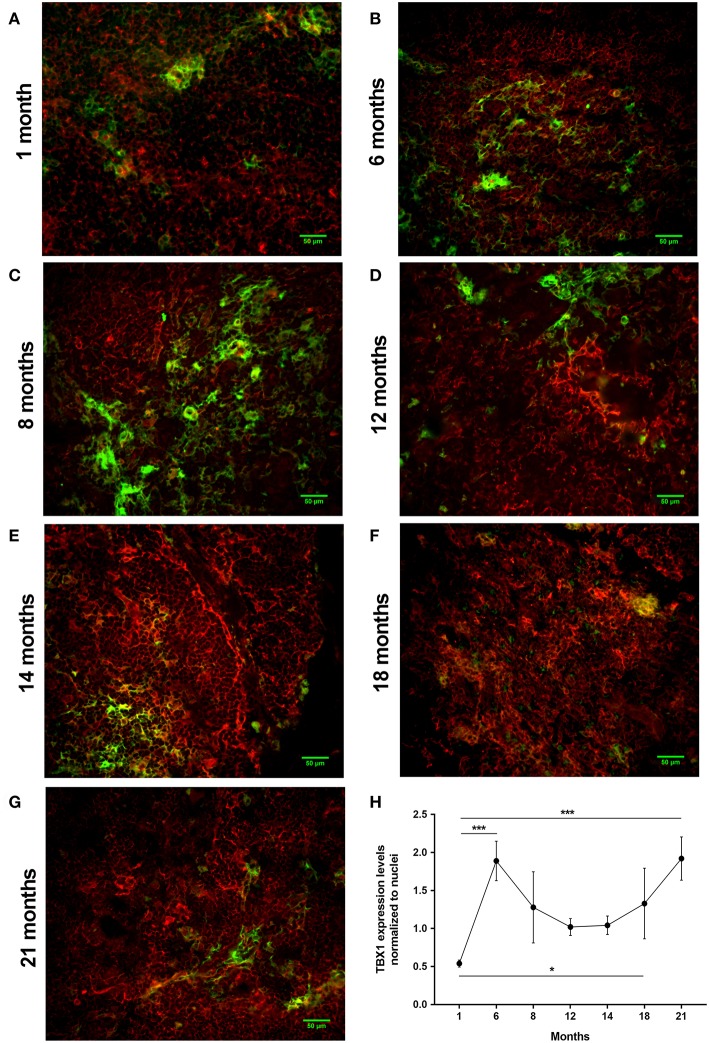
Kinetics of TBX-1 expression in the adult mouse thymus with age. Murine thymic frozen sections from different ages (1, 6, 8, 12, 14, 18, and 21 months) were evaluated by immune-fluorescent staining **(A–G)**, respectively. Epithelial network is shown in green (EpCAM1-FITC) while TBX-1 expression is shown in red (TBX1-Alexa555) fluorescence. Please note signs of degeneration (auto-fluorescence) at elevated ages. Please also note that TBX-1 staining pattern localizes to both nuclear and cytoplasmic bodies in accordance with The Human Protein Atlas: http://www.proteinatlas.org/ENSG00000184058-TBX1/cell. TBX-1 staining was normalized to DAPI nuclear counter-stain (not shown) and is presented as relative value **(H)**. Please note that relative TBX-1 expression shows a transient decrease at adult age (12 months of age). Significant differences are shown by asterisks (*p ≤ 0.05, ***p ≤ 0.001). Data were calculated from three slides, representative slide is shown. For exact numerical values and standard error of mean please refer to Supplementary Data Sheet.

Using a pilot set of mouse thymic cryosections of various ages (1, 6, 8, 12, 14, 18, and 21 months) we performed immune-fluorescent staining for beige adipose tissue-specific marker TBX-1 (Figures 3A–G). TBX-1 expression (in red) appears to persist throughout adulthood in the mouse similar to human above. EpCAM-1 staining (in green) shows medullary areas to demonstrate histological organization. Normalization to DAPI nuclear counterstain (in blue, not shown here) reveals that TBX-1 staining intensity transiently decreases at adult mid-term (12–14 months) to show rebound at senior ages (Figure 3H). In other terms: TBX-1 expression potentially appears to be bimodal in the mouse as well showing elevation at both young and senior ages with a transient in-between decrease during adulthood. Murine kinetics of TBX-1 expression resembles the previously shown human kinetics but with higher resolution in time. Please note medullary involution observed from 14 months of age onwards in line with our previous reports (4, 6).

#### Further Beige Adipocyte Markers Are Also Up-Regulated in Steroid-Induced Mouse TECs

**Figure 4:**
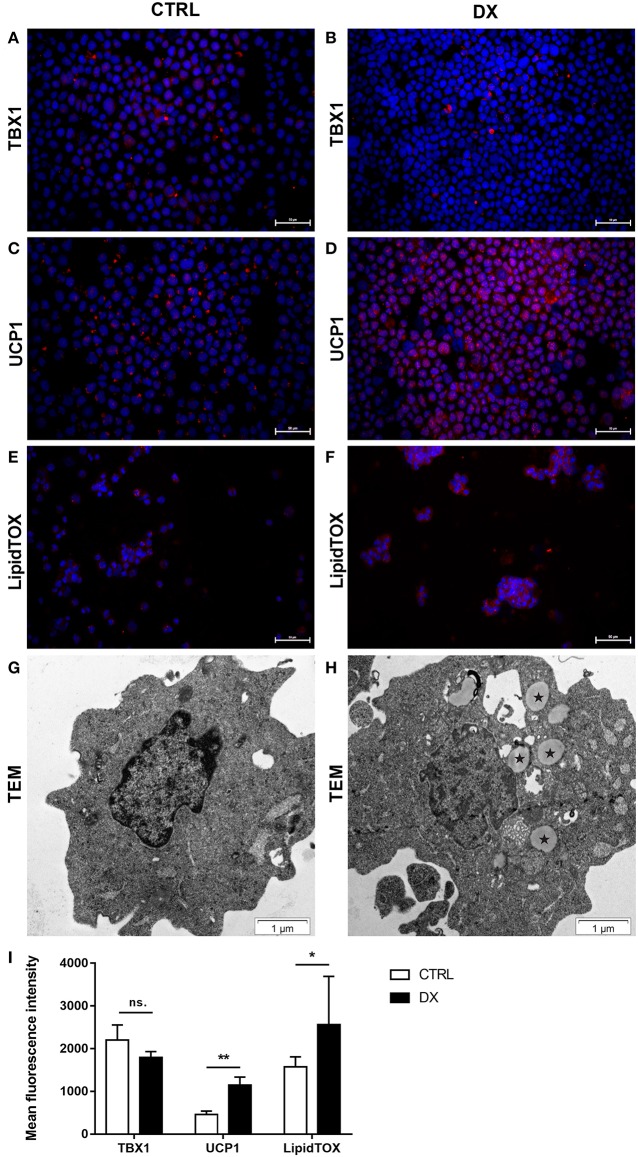
Beige adipocyte marker expression and lipid accumulation in steroid-induced mouse TECs. Cytospin slides of control (Ctrl) and steroid-induced (Dx) TEP1 cells were stained by immune-fluorescence. Beige-specific marker TBX-1 **(A,B)** and beige-indicative marker UCP-1 **(C,D)** was evaluated in red (Alexa555). Neutral lipid deposits were stained with LipidTOX Red dye **(E,F)**. DAPI staining was also applied as nuclear counter-stain. TBX-1, UCP-1, and LipidTOX staining relative to DAPI staining is also shown by histograms **(I)**. TBX-1 shows unaltered expression, while UCP-1 and lipid accumulation show significant increase following Dx-induction. Significant differences are shown by asterisks (*p ≤ 0.05, **p ≤ 0.01). Data were calculated from six slides, representative slide is shown. For exact numerical values and standard error of mean please refer to Supplementary Data Sheet. Ultrastructure of control (Ctrl) and steroid-induced (Dx) TEP1 cells was also evaluated by transmission electron microscopy (TEM) **(G,H)**, respectively. Asterisks (*) show intracellular multi-locular lipid deposits following Dx-induction.

As reported previously focusing on PPARgamma expression molecular level events are similar in the aging thymus and steroid-induced TECs in the mouse setting (4, 6). We have evaluated TEP1 mouse TECs for the expression of beige-specific and beige-indicative protein markers after Dx-treatment. TEP1 cells showed persistent, unchanged TBX-1 expression following Dx-treatment (Figures 4A,B,I). UCP-1 expression showed significant (*p* < 0.01) increase following Dx-treatment (Figures 4C,D,I). Lipid accumulation was also tested (LipidTox Red as above). The staining showed that Dx-treatment results in significant (*p* < 0.05) accumulation of neutral lipid deposits (Figures 4E,F,I) in accordance with our previous reports (4, 6). Ultra-structural imaging by TEM shows the appearance of multi-locular intracellular lipid deposits (indicated by asterisks) upon Dx-treatment, reminiscent of beige adipose tissue (Figures 4G,H).

### Steroid-Induced TECs Show Beige Adipocyte Metabolic Profile

There is a significant difference between white, brown and beige adipose tissue metabolic traits. In search of further evidence we have characterized the metabolic fingerprint of Dx-induced mouse TECs (TEP1).

**Figure 5:**
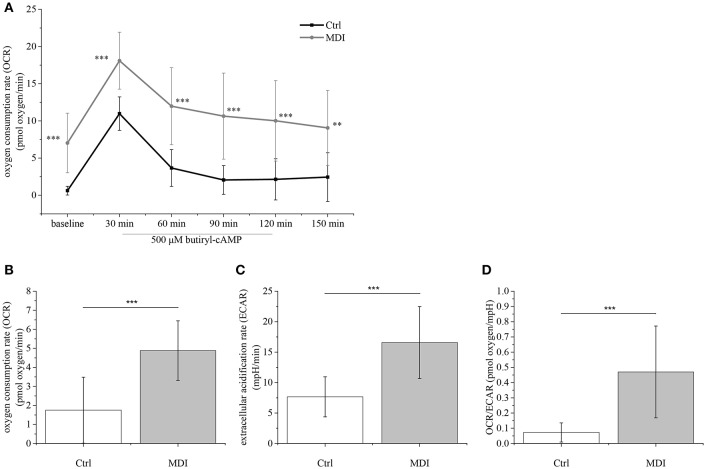
Metabolic parameters of steroid-induced TECs. Following pilot experiments, 15,000 cells / well were cultured for 9 days in MDI (or control) medium prior to Seahorse measurements. Baseline OCR was recorded followed by induction with cAMP (readings every 30 min) **(A)**. Cells were treated with oligomycin to show oligomycin-resistant respiration indicating mitochondrial inner membrane leakage **(B)**. ECAR was also determined **(C)** and the OCR/ECAR ratio was calculated **(D)**. Significant differences are shown by asterisks (**p ≤ 0.01, ***p ≤ 0.001). Data were calculated from forty measurements, mean is shown. For exact numerical values and standard error of mean please refer to Supplementary Data Sheet.

The metabolic fingerprint of TEP1 cells treated with Dx (as part of MDI differentiation medium) was assayed using the Seahorse platform (Figure 5). MDI cells showed significantly higher basal OCR values compared to control cells (*p* < 0.001) (Figure 5A). Of note cAMP-induced OCR was rapid (30 min post-treatment) and lasted shorter than in previous reports (32, 33). In line with elevated UCP-1 expression oligomycin-resistant respiration was significantly higher in MDI cells than in control cells (*p* < 0.001) (Figure 5B). Although we have recorded increased glycolysis marked by significantly increased ECAR values (*p* < 0.001) (Figure 5C), the significantly increased ratio of basal OCR and ECAR (*p* < 0.001) (Figure 5D) in MDI cells suggests their dependence on mitochondrial oxidation. Taking the observed increase in basal OCR value, OCR/ECAR ratio, cAMP-response, and oligomycin-resistant respiration into consideration, these suggest that MDI-differentiated TECs possess a beige metabolic fingerprint in accordance with the up-regulation of beige-specific and beige-indicative markers shown above.

### Aged and Steroid-Induced TECs Show Beige Adipocyte Gene Expression Profile

Adult human and mouse thymus sections showed similar histological changes with age. Likewise, mouse and human steroid-induced TECs were also similar by immune-fluorescent staining. Next, TECs enriched from adult mice or Dx-treated (murine or human) TECs were subjected to gene expression analysis.

**Figure 6:**
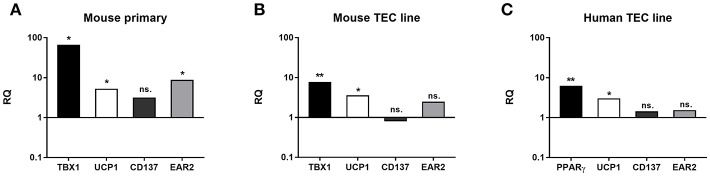
Beige adipocyte marker expression in aged or steroid-induced, mouse, and human TECs. Marker expression was evaluated by qRT-PCR from sorted TECs of mice **(A)**. TBX-1, UCP-1 and EAR-2 showed significant increase with age (1 m vs 12 m). CD137 remained unchanged. Marker expression was evaluated by qRT-PCR in Dx-induced mouse TEP1 cells **(B)** and human 1889c cells **(C)**, respectively. In mouse TEP1 cells TBX-1 and UCP-1 showed significant increase with Dx-induction. CD137 and EAR-2 did not present significant difference. In human 1889c cells PPAR-gamma and UCP-1 showed significant increase with Dx-induction. CD137 and EAR-2 did not present significant difference. Relative quantity values (RQ) are shown where Y = 1 represents young adult **(A)** or control expression levels **(B,C)**, respectively. Significant differences are shown by asterisks (*p ≤ 0.05, **p ≤ 0.01). Please not that Y-axis is logarithmic. For exact numerical values and standard error of mean please refer to Supplementary Data Sheet containing both RQ and Ct/SD values for all experiments and target genes.

Changes in gene expression were further tested at the mRNA level in EpCAM1-enriched primary murine thymic epithelial cells from senior adult age (12 m) and steroid-induced TEP1 or 1889c cells for beige-specific (TBX1) and beige-indicative genes (UCP1, CD137, EAR2) (21–26). Enriched cells showed the up-regulation of both beige-specific and beige-indicative genes with age (1 vs. 12 months) as TBX1, UCP1, and EAR2 all showed significant elevation (*p* < 0.05 for all, Figure 6A), while CD137 activity remained unchanged. Gene expression analysis of mouse TEC line following Dx-treatment showed a similar tendency. as significant increase of TBX1 and UCP1 expression was detected (*p* < 0.01 and *p* < 0.05, respectively, Figure 6B), while CD137 and EAR2 were not altered. Likewise, the steroid-induced human TEC line showed significantly increased PPAR-gamma expression (*p* < 0.01) as reported previously for mouse TECs (4) and also significant increase of UCP-1 expression (*p* < 0.05) (Figure 6C), while CD137 and EAR2 remained identical. Please note the harmony of *in vivo* and *in vitro* data in both mouse and human species supporting our observations.

### Steroid-Induced TECs Show Beige Adipocyte miRNA Profile

There is a significant difference between white, brown and beige adipose tissue miRNA profile. Seeking further evidence we have characterized the miRNA profile of Dx-induced human TECs (1889c).

**Figure 7:**
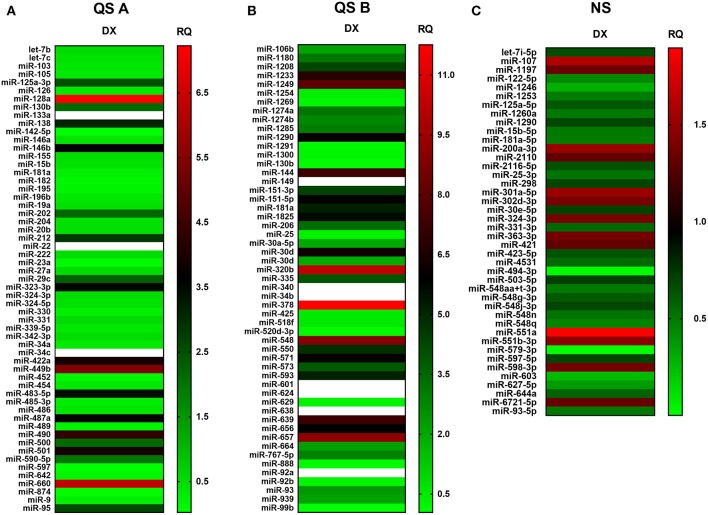
miRNA profile of steroid-induced TECs. miRNA profile of Dx-treated human 1889c cells measured by Quantstudio 12K Flex Pool A **(A)**, Pool B **(B)** and NanoString nCounter SPRINT Profiler **(C)**. Heat-map representation shows miRNA species with most significant changes in copy number. Relative quantity values are presented (ctrl = 1). Up-regulation of miRNA species is shown in red while down-regulation is shown in green. Out-of-scale RQ values are shown in white. Pilot study is shown, for exact numerical values please refer to Supplementary Data Sheet.

We have elaborated two distinct platforms (Figure 7) for complete human miRNome analysis. For both platforms increased copy numbers are shown in red, while decreased copy numbers are shown in green (heat map). QuantStudio miRNA (QS) panels (A and B, Figures 7A,B) evaluate 768 miRNA entities, while the NanoString (NS) cartridge measures copy numbers of 880 miRNA entries (Figure 7C). Of note QS is amplification- (PCR) based while NS is amplification free. QS provides enhanced sensitivity, NS ensures unmatched signal-to-noise ratio. Accordingly, QS identified more miRNA species with occasional out-of-scale activities (shown in white) while NS recognized less species with a compressed scale of activities relative to QS. An overlap of the recognized miRNA species identified by at least one platform or evaluated by both platforms similarly is summarized by Table 2. The table connects the identified miRNA species with context-relevant function based on literature search. Of note, several of the recognized species have relevance to thymus senescence with special focus on adipose tissue development, epithelial-to-mesenchymal transition, cell proliferation and senescence.

**Table 2:** Overlap of QS- and NS-based miRNA results with functional and literature annotation.

**Name**	**Up/down regulation**	**QS/NS**	**Function**	**References**
miR-103a-3p		QS A	Inactivation upregulates insulin receptors in adipocytes	(34)
miR-105-5p		QS A, NS	Epithelial to mesenchymal transition	(35)
miR-106b		QS B	Beige adipose tissue regulator	(36)
miR-1208		QS B	Targets TGFB2 (involved in adipose tissue development)	(37)
miR-1246		NS	Promotes cell proliferation	(38)
miR-125a-3p		QS A	Tissue-specific senescence	(39)
miR-125a-5p		QS A, NS	Regulation of epithelial cell differentiation	(40)
miR-126-3p		QS A	Insulin/IGF1 signaling pathway	(41)
miR-1274a		QS B	Potential biomarker for Alzheimer's Disease	(42)
miR-1274b		QS B	Potential biomarker for Alzheimer's Disease	(42)
miR-128a-3p		QS A	Regulatory effect on PPARg	(43)
miR-138-5p		QS A	Negative regulation of apoptosis	(44)
miR-15b-5p		QS A, NS	Characteristic of senescent cell derived EVs	(36, 45)
miR-155		QS A	Induces brown adipocyte differentiation from white adipocytes	(46)
miR-181a-5p		QS A, NS	Stress-related thymic involution	(47)
miR-1825		QS B	Lipid signaling	(48, 49)
miR-200a-3p		NS	Regulates epithelial cell transformation (EMT and MET)	(50)
miR-2110		NS	Cellular development, cell-mediated immune response	(51)
miR-2116-5p		NS	Regulatory function in colorectal cancer	(52)
miR-25-3p		QS A, B, NS	Modulator of the Wnt pathway	(47)
miR-27a		QS A, B	Negative regulator in beige adipose tissue	(36, 53)
miR-301a-5p		NS	Role in adipogenesis	(54)
miR-30d		QS B	Upregulation in adipose tissue	(55)
miR-323-3p		QS A	Regulation of senescence through IGF signaling pathway	(56)
miR-331-3p		NS, QS A	Induces senescence and cell cycle arrest	(57, 58)
miR-421		NS	Upregulation modulates oxidant stress and lipid metabolism	(59)
miR-425-5p		QS A, B	Inhibits differentiation and proliferation of preadipocytes	(40)
miR-4531		NS	Involved in type 1 diabetes mellitus	(60)
miR-520d-3p		NS, QS B	Regulatory function in colorectal cancer	(52)
miR-548q		NS	Possible biomarker of nasopharyngeal carcinoma	(61)
miR-550a		QS B	Adipogenic differentiation	(62)
miR-597-5p		NS, QS A	Drives EMT	(63)
miR-657		QS B	Regulates IL-37/NF-κB signaling	(64)
miR-6721-5p		NS	Unknown	
miR-888		QS B, NS	Downregulates E-cadherin	(65)
miR-92a-3p		QS A, NS	Replicative and organismal human aging	(36)
miR-92b-3p		QS B, NS	Regulation of lipid deposition	(66, 67)
miR-939-5p		QS B, NS	Inhibits cell proliferation	(68)
miR-99b		QS A, B, NS	Regulates epithelial cell differentiation	(36, 69, 70)

## Discussion

### Thymic Tissue Samples and Steroid-Induced TECs Show Beige Adipocyte Markers

TBX-1 has been extensively studied for its role in the thymic context during embryonic organogenesis, but not in the adult thymus undergoing adipose involution (11–15). Using human and mouse thymus sections we show that TBX-1 expression persists throughout adulthood with a transient decrease in expression (23 years of age in human and 12 months of age in mouse) based on our pilot studies. This persistence of thymic TBX-1 expression raises the possibility of an alternative role in adulthood. This hypothesis is supported by reports showing that (1) once the thymus has been formed TBX-1 suppresses FoxN1 (key transcription factor of thymic epithelial identity) and (2) TBX-1 has a key and specific role in beige adipose tissue development (17–21). This plausible connection is supported by our results as further beige-indicative markers (UCP-1, EAR2) show increasing mRNA levels with age. This is in harmony with the fact that the thymus resides in the mediastinum and secretes FGF21, both reported to promote the emergence of beige adipose tissue (25). The *in vitro* model system of aging (Dx-treated mouse TEP1 or human 1889c cells) show similar molecular and cellular changes. Immune-fluorescent histology shows the presence of TBX-1 both in control conditions and following Dx-treatment. UCP-1 protein expression, on the other hand, significantly increases following Dx-treatment. Protein level data are in accordance with mRNA results as both TBX-1 and UCP-1 showed an increase following Dx-treatment (both in mouse and human). The above molecular changes are accompanied by evident phenotypical changes: the appearance of typical intracellular multi-locular neutral lipid deposits (characteristic to brown/beige adipose tissue) as shown by LipidTox staining and transmission electron microscopy. Taken together, these data suggest that thymic adipose tissue emerging with senescence and modeled by steroid-induced TECs show beige adipocyte features.

### Steroid-Induced TECs Show Beige Adipocyte Metabolic Profile

There is profound difference between white and brown/beige adipose tissues with respect to metabolic traits (23). Basal respiration (OCR) is significantly lower in white fat cells than in brown/beige fat cells. Also, UCP-mediated uncoupled respiration rate (resistant to inhibition by oligomycin) is characteristic to brown/beige fat cells and not observed in white fat cells (32). Furthermore, in brown/beige fat cells cAMP-induced mitochondrial oxidation is elevated compared to white fat cells (33). Having analyzed these metabolic parameters, our data suggest that adipocyte differentiation in our model system shows beige bias as indicated by elevated basal OCR, increased OCR/ECAR ratio, enhanced cAMP-response and oligomycin-resistant respiration. Our metabolic readouts are in accordance with the recorded beige adipose tissue markers, morphological characteristics and gene expression profiles.

### Steroid-Induced TECs Show Beige Adipocyte miRNA Profile

Unbiased dual platform complete miRNome analysis identified a number of context-relevant miRNA copy number alterations. Of note, miR-27a and miR-106b are beige adipose tissue regulators and miR-155 is an inhibitor of brown/beige adipose tissue formation (36, 46, 53). From a broad pan-adipocyte perspective, miR-128a-3p, miR-1825, miR-301a-5p, miR-30d, miR-425-5p, miR-550a, and miR92b-3p also influence adipose tissue formation and show changes in the current experimental setting (40, 43, 48, 49, 54, 55, 62, 66, 67). Furthermore, a cornerstone of thymus adipose involution: epithelial-to-mesenchymal transition (EMT), operates via miR-105-5p, miR-200a-3p, miR-597-5p, miR-888, and miR-99b, all demonstrating changes in copy number in steroid-induced TECs (35, 36, 50, 63, 65, 69, 70). Taking a final expansion of interest, from a senescent perspective miR-125a-3p, miR-125a-5p, miR-15b-5p, miR-181a-5p, miR-323-3p, and miR-331-3p affect cellular / tissue level senescence with focus on the thymus and also show significant changes (36, 39, 40, 45, 47, 56–58). In summary, steroid-treatment in TECs affects the same miRNA species that were reported in connection with senescence-related thymus adipose involution that apparently yields beige adipose tissue.

### Expanding Overlap Between Metabolism and Immunity

Overlap of metabolism and immunity has already been raised decades ago, and this inter-disciplinary field has recently become a prominent research area. For example, both previous and recent papers discussed overlap between the neuroendocrine and immune systems with regard to melatonin (71–73). Melatonin—mainly produced by the pineal gland, but also expressed by the thymus in small amounts—has been reported to have an immune-modulatory effect, enhancing immune functions with Th1 bias. Accordingly, anti-viral and anti-cancer defense is boosted by melatonin and age-related loss of melatonin production partly explains elevated incidence of infection and cancer observed with senescence. Protection from cancer metastasis development in the central nervous system (CNS) implies proper blood-brain barrier (BBB) function (74–76). BBB function, CNS function and immune status are all controlled by metabolic interplays involving small molecules such as lactate. Local tissue lactate concentration has been reported to have important role in immune regulation, its accumulation promoting autoimmune reactions (77, 78). Intercellular immune modulatory signals triggered by metabolically active small molecules are transmitted in cells through signaling pathways. An important pathway connecting metabolism and immunity utilizes mechanistic target of rapamycin (mTOR). It was shown that mTOR senses certain small nutrients (amino acids) and thus affects immune tolerance through regulatory T-cells (79, 80). Mammalian immunity heavily relies on both the innate and the adaptive branch. Within innate immunity macrophages have an important role in connecting metabolism and immunity. It has also been reported that carbohydrate metabolism significantly affects inflammation via macrophages (81).

Carbohydrates are also basic metabolic fuels. The mammalian immune system is a costly defense system with regard to T-cell development and selection taking place in the thymus, where approx. Ninety-five percent of developing thymocytes are deleted being useless or potentially autoimmune. However, the adaptive branch heavily relies on the constant supply of fresh naïve and scrupulously selected T-cells to prevent infection, cancer and autoimmunity from developing. Severe negative imbalance in energy expenditure (due to fasting or malnutrition) has long been known to hamper thymus function and immunity (82). In contrast, currently, global human population is more threatened by obesity than fasting along with its reported negative effects on thymus function (1, 83). Fashionable countermeasures of obesity include e.g., applying diet to induce ketosis. Ketosis has been reported to enhance FGF21 secretion, known to promote white adipose tissue browning especially in the mediastinal context, where the thymus also resides (25, 84). Further options of white adipose tissue browning include interventions e.g., irisin (exercise hormone) treatment (32). However, since irisin promotes beige adipose tissue development it may also impair thymus function via promoting adipose involution identical to thymus senescence.

Our study highlights another potential intersection of immunity and metabolism via the dual role of TBX-1 during thymus development and senescence. TBX-1 shows bimodal expression (high expression in early and late ages, with a transient decrease in-between) in both mouse and human. It is conceivable that TBX-1 plays a role in thymus organogenesis early on (early “immune” peak) and thymic adipose involution later on (late “metabolic” peak). This dualism may be unique to the thymus due to the observed “beige” adipose involution process.

With senescence the thymus suffers adipose involution. Impaired thymic niche leads to decreased naïve T-cell output. This in turn weakens T cell-mediated anti-viral and anti-cancer defense, and elevates the chances of autoimmune disorders due to dysfunctional T-cell selection. Therefore, thymic adipose tissue emerging with age impairs immune homeostasis and the maintenance of tolerance. Our results indicate that thymic adipose tissue shows “beige” characteristics by molecular, cellular and metabolic profiling. Our research contributes to the breadth of overlap between metabolism and immune homeostasis.

## Data Availability

All datasets generated for this study are included in the manuscript and/or the Supplementary Files.

## Ethics Statement

Mice were kept in the Laboratory Animal Core Facility of the University. Experimental procedures were carried out according to the 1988/XXVIII act of the Hungarian Parliament on Animal Protection (243/1988) which complies with recommendations of the Helsinki Declaration. All animal experiments were performed with the consent of the Ethics Committee on Animal Research of the University (ref. no.: #BA02/2000-46/2016). Formalin-fixed, paraffin-embedded (FFPE) human thymus samples from 18, 23, 42, 44, and 58 years of age were provided by the Department of Pathology, Faculty of Medicine, University of Pecs, Hungary. Experiments involving human samples were performed with the consent of the Regional and Local Ethics Committee of Clinical Centre of the University (ref. no.: 6069/2016) according to their guidelines. All subjects gave written informed consent in accordance with the Declaration of Helsinki.

## Author Contributions

KB performed most histological, molecular biology, and statistics work in the project and was involved in manuscript preparation. DE performed all human IHC work. AP and PB were responsible for preparative Seahorse measurements. KG performed statistical analysis. DB was involved in experiments performed on human 1889c cells. JP was involved in planning experiments and manuscript preparation as well as local supervision of respective department. KK was involved in histological, molecular biology, and statistics work, also in planning experiments and manuscript preparation, and supervised the project.

### Conflict of Interest Statement

The authors declare that the research was conducted in the absence of any commercial or financial relationships that could be construed as a potential conflict of interest.
