# Supplementary material for: “Beige” Cross Talk Between the Immune System and Metabolism
Source: Front Endocrinol (Lausanne). 2019 Jun 18;10:369. doi: 10.3389/fendo.2019.00369 (PMC6591453; doi:10.3389/fendo.2019.00369)
Supplement: Supplementary file 1 [file Table_1.DOCX]

**SUPPLEMENTARY DATA SHEET**

**Numerical data of figures**

**Figure 1.**

Immune histology staining for TBX1 on human tissue (Tbx1 expression levels relative to nuclei using positive pixel counts)

| **Age group** | **Mean** | **SD** |
| --- | --- | --- |
| 18 years | 0.475 | 0.047 |
| 23 years | 0.266 | 0.056 |
| 42 years | 0.581 | 0.063 |
| 44 years | 0.604 | 0.050 |
| 58 years | 0.624 | 0.100 |

| **Tests of Normality** | | | | | | |
| --- | --- | --- | --- | --- | --- | --- |
| **Age** | Kolmogorov-Smirnov^a^ | | | Shapiro-Wilk | | |
|  | Statistic | df | Sig. | Statistic | df | Sig. |
| 18 years | 0.177 | 3 | . | 10.000 | 3 | 0.962 |
| 23 years | 0.250 | 3 | . | 0.967 | 3 | 0.651 |
| 42 years | 0.209 | 3 | . | 0.992 | 3 | 0.825 |
| 44 years | 0.208 | 3 | . | 0.992 | 3 | 0.827 |
| 58 years | 0.269 | 3 | . | 0.950 | 3 | 0.569 |
| a. Lilliefors Significance Correction | | | | | | |

|  | | | |
| --- | --- | --- | --- |
| **Test of Homogeneity of Variances** | | | |
| Levene Statistic | df1 | df2 | Sig. |
| 0.773 | 4 | 11 | 0.565 |
|  |  |  |  |

|  | | | | | |
| --- | --- | --- | --- | --- | --- |
| **ANOVA** | | | | | |
|  | Sum of Squares | df | Mean Square | F | Sig. |
| Between Groups | 0.319 | 4 | 0.080 | 180.585 | **0.000** |
| Within Groups | 0.047 | 11 | 0.004 |  |  |
| Total | 0.366 | 15 |  |  |  |

|  | | | | | | |
| --- | --- | --- | --- | --- | --- | --- |
| **Multiple Comparisons** | | | | | | |
| Tukey HSD | | | | | | |
| (I) Group2 | (J) Group2 | Mean Difference (I-J) | Std. Error | Sig. | 95% Confidence Interval | |
|  |  |  |  |  | Lower Bound | Upper Bound |
| 18 | 23 | 0.209786^*^ | 0.050004 | **0.010** | 0.04807 | 0.37150 |
|  | 42 | -0.105292 | 0.053457 | 0.340 | -0.27817 | 0.06759 |
|  | 44 | -0.128390 | 0.053457 | 0.186 | -0.30127 | 0.04449 |
|  | 58 | -0.148051 | 0.053457 | 0.106 | -0.32093 | 0.02483 |
| 23 | 18 | -0.209786^*^ | 0.050004 | **0.010** | -0.37150 | -0.04807 |
|  | 42 | -0.315078^*^ | 0.050004 | **0.000** | -0.47679 | -0.15336 |
|  | 44 | -0.338176^*^ | 0.050004 | **0.000** | -0.49989 | -0.17646 |
|  | 58 | -0.357837^*^ | 0.050004 | **0.000** | -0.51955 | -0.19612 |
| 42 | 18 | 0.105292 | 0.053457 | 0.340 | -0.06759 | 0.27817 |
|  | 23 | 0.315078^*^ | 0.050004 | **0.000** | 0.15336 | 0.47679 |
|  | 44 | -0.023097 | 0.053457 | 0.992 | -0.19598 | 0.14978 |
|  | 58 | -0.042759 | 0.053457 | 0.925 | -0.21564 | 0.13012 |
| 44 | 18 | 0.128390 | 0.053457 | 0.186 | -0.04449 | 0.30127 |
|  | 23 | 0.338176^*^ | 0.050004 | **0.000** | 0.17646 | 0.49989 |
|  | 42 | 0.023097 | 0.053457 | 0.992 | -0.14978 | 0.19598 |
|  | 58 | -0.019662 | 0.053457 | 0.995 | -0.19254 | 0.15322 |
| 58 | 18 | 0.148051 | 0.053457 | 0.106 | -0.02483 | 0.32093 |
|  | 23 | 0.357837^*^ | 0.050004 | **0.000** | 0.19612 | 0.51955 |
|  | 42 | 0.042759 | 0.053457 | 0.925 | -0.13012 | 0.21564 |
|  | 44 | 0.019662 | 0.053457 | 0.995 | -0.15322 | 0.19254 |
| *. The mean difference is significant at the 0.05 level. | | | | | | |

**Figure 2.**

Immune fluorescent staining for lipid markers on human 1889c cells (mean fluorescence intensity)

|  | **Ctrl** | | **Dx** | |
| --- | --- | --- | --- | --- |
|  | **Mean** | **SD** | **Mean** | **SD** |
| **PPARg** | 152.300 | 26.630 | 472.600 | 74.040 |
| **Tbx1** | 481.400 | 38.300 | 646.600 | 28.540 |
| **Ucp1** | 589.000 | 316.100 | 909.700 | 194.200 |
| **LipidTOX** | 145.300 | 17.020 | 324.100 | 44.850 |

**Figure 3.**

Immune fluorescent staining for TBX1 on mouse tissue ((Tbx1 expression levels relative to nuclei using mean fluorescent intensity values)

| **Age group** | **Mean** | **SD** |
| --- | --- | --- |
| 1 month | 0.540 | 0.045 |
| 6 months | 1.890 | 0.260 |
| 8 months | 1.279 | 0.468 |
| 12 months | 1.020 | 0.111 |
| 14 months | 1.043 | 0.122 |
| 18 months | 1.329 | 0.464 |
| 21 months | 1.921 | 0.285 |

| **Tests of Normality** | | | | | | |
| --- | --- | --- | --- | --- | --- | --- |
|  | Kolmogorov-Smirnov^a^ | | | Shapiro-Wilk | | |
|  | Statistic | df | Sig. | Statistic | df | Sig. |
| 1 month | 0.310 | 3 | . | 0.899 | 3 | 0.382 |
| 6 months | 0.272 | 3 | . | 0.947 | 3 | 0.555 |
| 8 months | 0.342 | 3 | . | 0.844 | 3 | 0.225 |
| 12 months | 0.385 | 3 | . | 0.750 | 3 | **0.000** |
| 14 months | 0.320 | 3 | . | 0.883 | 3 | 0.333 |
| 18 months | 0.290 | 3 | . | 0.926 | 3 | 0.475 |
| 21 months | 0.177 | 3 | . | 10.000 | 3 | 0.962 |
| a. Lilliefors Significance Correction | | | | | | |

**Hypothesis test summary**

|  | **Null Hypothesis** | **Test** | **Sig.** | **Decision** |
| --- | --- | --- | --- | --- |
| **1** | The distribution of months is the same across categories of Groupvar. | Independent Samples  Kruskal-Wallis test | 0.008 | Reject the null hypothesis. |

Asympthotic significances are displayed. The significance level is 0.05.

Each node shows the sample average rank of Goupvar.

| **Sample1- Sample 2 (Months)** | **Test Statistic** | **Std. Error** | **Std. Test Statistic** | **Sig.** | **Adj. Sig.** |
| --- | --- | --- | --- | --- | --- |
| **1-12** | -7.167 | 5.399 | -1.327 | 0.184 | 1.000 |
| **1-14** | -8.000 | 4.999 | -1.600 | 0.110 | 1.000 |
| **1-8** | -10.167 | 5.399 | -1.883 | 0.060 | 1.000 |
| **1-18** | -11.500 | 4.999 | -2.301 | **0.021** | 0.450 |
| **1-6** | -18.167 | 5.399 | -3.365 | **0.001** | 0.016 |
| **1-21** | -18.500 | 5.399 | -3.426 | **0.001** | 0.013 |
| **12-14** | -0.833 | 5.399 | -0.154 | 0.877 | 1.000 |
| **12-8** | 3.000 | 5.772 | 0.520 | 0.603 | 1.000 |
| **12-18** | -4.333 | 5.399 | -0.803 | 0.422 | 1.000 |
| **12-6** | 11.000 | 5.772 | 1.906 | 0.057 | 1.000 |
| **12-21** | -11.333 | 5.772 | -1.963 | 0.050 | 1.000 |
| **14-8** | 2.167 | 5.399 | 0.401 | 0.688 | 1.000 |
| **14-18** | -3.500 | 4.999 | -0.700 | 0.484 | 1.000 |
| **14-6** | 10.167 | 5.399 | 1.833 | 0.060 | 1.000 |
| **14-21** | -10.500 | 5.399 | -1.945 | 0.052 | 1.000 |
| **8-18** | -1.333 | 5.399 | -0.247 | 0.805 | 1.000 |
| **8-6** | 8.000 | 5.772 | 1.386 | 0.166 | 1.000 |
| **8-21** | -8.333 | 5.772 | -1.444 | 0.149 | 1.000 |
| **18-6** | 6.667 | 5.399 | 1.235 | 0.217 | 1.000 |
| **18-21** | -7.000 | 5.399 | -1.296 | 0.195 | 1.000 |
| **6-21** | -0.333 | 5.772 | -0.058 | 0.954 | 1.000 |

Each row tests the null hypothesis that the Sample 1 and Sample 2 distributions are the same. Asymptotic significances (2-sided tests) are displayed. The significance level is 0.05.

**Figure 4.**

Immune fluorescent staining for lipid markers on mouse TEP1 cells (mean fluorescence intensity)

|  | **Ctrl** | | **Dx** | |
| --- | --- | --- | --- | --- |
|  | **Mean** | **SD** | **Mean** | **SD** |
| **Tbx1** | 2202.000 | 353.300 | 1790.000 | 140.700 |
| **Ucp1** | 460.600 | 77.980 | 1144.000 | 190.700 |
| **LipidTOX** | 1573.000 | 236.500 | 2560.000 | 1132.000 |

**Figure 5.**

Oxygen consumption rate (OCR) of mouse TEP1 cells

|  | **Ctrl** | | **MDI** | |
| --- | --- | --- | --- | --- |
|  | **Mean** | **SD** | **Mean** | **SD** |
| baseline | 0.615 | 0.588 | 7.024 | 4.001 |
| cAMP 30 min | 10.977 | 2.249 | 18.097 | 3.832 |
| cAMP 60 min | 3.664 | 2.475 | 11.978 | 5.176 |
| cAMP 90 min | 2.053 | 1.936 | 10.639 | 5.791 |
| cAMP 120 min | 2.138 | 2.773 | 10.012 | 5.413 |
| cAMP 150 min | 2.443 | 3.276 | 9.056 | 5.041 |

Oxygen consumption rate (OCR) (pmol oxygen/min) of mouse TEP1 cells

| **Ctrl** | | **MDI** | |
| --- | --- | --- | --- |
| **Mean** | **SD** | **Mean** | **SD** |
| 1.751 | 1.733 | 4.880 | 1.565 |

Extracellular acidification rate (ECAR) (mpH/min) of mouse TEP1 cells

| **Ctrl** | | **MDI** | |
| --- | --- | --- | --- |
| **Mean** | **SD** | **Mean** | **SD** |
| 7.664 | 3.288 | 16.574 | 5.901 |

OCR/ECAR ratio (pmol oxygen/mpH) of mouse TEP1 cells

| **Ctrl** | | **MDI** | |
| --- | --- | --- | --- |
| **Mean** | **SD** | **Mean** | **SD** |
| 0.072 | 0.063 | 0.470 | 0.302 |

**Figure 6**.

Target gene expression values (RQ and Ct/SD) following normalization to

housekeeping gene (actin)

Fold change (RQ) gene expression by qPCR during mouse thymic aging

| **Tbx1** | **Ucp1** | **CD137** | **Ear2** |
| --- | --- | --- | --- |
| 64.36 | 5.11 | 3.07 | 8.47 |

Absolute gene expression (Ct/SD, young vs. old) by qPCR during mouse thymic aging

| **Tbx1** | **Ucp1** | **CD137** | **Ear2** |
| --- | --- | --- | --- |
| 12.29/3.65 vs.  6.28/0.71 | 11.80/1.22 vs.  9.45/0.79 | 6.54/2.86 vs.  4.92/2.90 | 11.68/0.80 vs.  8.60/1.67 |

Fold change (RQ) gene expression by qPCR following Dx treatment of mouse TEP1 cells

| **Tbx1** | **Ucp1** | **CD137** | **Ear2** |
| --- | --- | --- | --- |
| 7.41 | 3.48 | 0.84 | 2.39 |

Absolute gene expression (Ct/SD, control vs. DX) by qPCR following

Dx treatment of mouse TEP1 cells

| **Tbx1** | **Ucp1** | **CD137** | **Ear2** |
| --- | --- | --- | --- |
| 15.56/1.38 vs.  12.67/1.12 | 17.94/0.56 vs.  16.14/0.38 | 15.44/1.99 vs.  15.70/0.74 | 20.22/0.75 vs.  18.96/1.70 |

Fold change (RQ) gene expression by qPCR following Dx treatment of human 1889c cells

| **PPARg** | **Ucp1** | **CD137** | **Ear2** |
| --- | --- | --- | --- |
| 6.00 | 2.95 | 1.32 | 1.49 |

Absolute gene expression (Ct/SD, control vs. DX) by qPCR following

Dx treatment of human 1889c cells

| **PPARg** | **Ucp1** | **CD137** | **Ear2** |
| --- | --- | --- | --- |
| 9.91/0.68 vs.  7.32/1.04 | 14.22/0.45 vs.  12.66/0.75 | 8.85/2.45 vs.  8.45/1.35 | 4.62/0.41 vs.  4.04/0.36 |

**Figure 7.**

A. miRNA expression of 1889c Dx treatment detected by Quantstudio 12K Flex Pool A (ctrl=1)

| **Target** | **RQ** |
| --- | --- |
| hsa-miR-133a-002246 | 1765601.500 |
| hsa-miR-22-000398 | 66.360 |
| hsa-miR-34c-000428 | 29.014 |
| hsa-miR-128a-002216 | 7.226 |
| hsa-miR-660-001515 | 6.355 |
| hsa-miR-449b-001608 | 5.426 |
| mmu-miR-615-001960 | 4.703 |
| hsa-miR-490-001037 | 4.331 |
| hsa-miR-422a-002297 | 3.936 |
| hsa-miR-501-001047 | 3.882 |
| hsa-miR-146b-001097 | 3.605 |
| hsa-miR-487a-001279 | 3.591 |
| hsa-miR-323-3p-002227 | 3.527 |
| hsa-miR-483-5p-002338 | 3.417 |
| hsa-miR-138-002284 | 3.113 |
| hsa-miR-212-000515 | 2.796 |
| hsa-miR-95-000433 | 2.590 |
| hsa-miR-125a-3p-002199 | 2.515 |
| hsa-miR-29c-000587 | 2.271 |
| hsa-miR-202-002363 | 2.160 |
| hsa-miR-500-002428 | 2.107 |
| hsa-miR-130b-000456 | 2.090 |
| hsa-miR-590-5p-001984 | 1.960 |
| hsa-miR-21-000397 | 1.881 |
| hsa-miR-671-3p-002322 | 1.826 |
| mmu-miR-140-001187 | 1.825 |
| hsa-miR-147b-002262 | 1.806 |
| hsa-miR-744-002324 | 1.781 |
| hsa-miR-100-000437 | 1.576 |
| hsa-miR-223-002295 | 1.428 |
| hsa-miR-148b-000471 | 1.423 |
| hsa-miR-19b-000396 | 1.405 |
| hsa-miR-509-5p-002235 | 1.378 |
| hsa-miR-339-3p-002184 | 1.336 |
| hsa-miR-20a-000580 | 1.292 |
| hsa-let-7f-000382 | 1.290 |
| hsa-miR-221-000524 | 1.263 |
| hsa-miR-625-002431 | 1.230 |
| mmu-miR-491-001630 | 1.217 |
| hsa-miR-132-000457 | 1.175 |
| hsa-miR-99a-000435 | 1.135 |
| hsa-miR-636-002088 | 1.105 |
| hsa-miR-494-002365 | 1.103 |
| hsa-miR-210-000512 | 1.094 |
| hsa-miR-532-3p-002355 | 1.016 |
| hsa-miR-29a-002112 | 1.013 |
| hsa-miR-17-002308 | 0.988 |
| hsa-miR-140-3p-002234 | 0.974 |
| hsa-miR-26a-000405 | 0.959 |
| hsa-miR-191-002299 | 0.946 |
| hsa-miR-16-000391 | 0.938 |
| hsa-miR-193b-002367 | 0.937 |
| hsa-miR-24-000402 | 0.931 |
| hsa-miR-501-3p-002435 | 0.925 |
| hsa-miR-28-000411 | 0.912 |
| hsa-miR-423-5p-002340 | 0.903 |
| hsa-miR-186-002285 | 0.889 |
| hsa-miR-106a-002169 | 0.881 |
| hsa-miR-574-3p-002349 | 0.864 |
| hsa-miR-30c-000419 | 0.858 |
| hsa-miR-125b-000449 | 0.855 |
| hsa-miR-181c-000482 | 0.848 |
| mmu-miR-93-001090 | 0.824 |
| hsa-miR-328-000543 | 0.806 |
| hsa-miR-362-001273 | 0.806 |
| hsa-miR-320-002277 | 0.805 |
| hsa-miR-130a-000454 | 0.798 |
| hsa-miR-28-3p-002446 | 0.788 |
| hsa-miR-183-002269 | 0.784 |
| hsa-miR-31-002279 | 0.760 |
| hsa-miR-365-001020 | 0.757 |
| hsa-miR-26b-000407 | 0.753 |
| hsa-miR-885-5p-002296 | 0.748 |
| hsa-miR-30b-000602 | 0.747 |
| hsa-miR-522-002413 | 0.743 |
| hsa-miR-331-5p-002233 | 0.742 |
| hsa-miR-139-5p-002289 | 0.723 |
| hsa-let-7a-000377 | 0.711 |
| hsa-miR-425-5p-001516 | 0.692 |
| hsa-miR-197-000497 | 0.692 |
| hsa-miR-193a-5p-002281 | 0.688 |
| hsa-miR-618-001593 | 0.685 |
| hsa-miR-532-001518 | 0.683 |
| hsa-let-7g-002282 | 0.666 |
| hsa-let-7d-002283 | 0.649 |
| hsa-let-7e-002406 | 0.641 |
| hsa-miR-192-000491 | 0.640 |
| hsa-miR-484-001821 | 0.627 |
| hsa-miR-27b-000409 | 0.624 |
| hsa-miR-98-000577 | 0.619 |
| hsa-miR-486-3p-002093 | 0.615 |
| hsa-miR-92a-000431 | 0.601 |
| hsa-miR-25-000403 | 0.597 |
| mmu-miR-374-5p-001319 | 0.585 |
| hsa-miR-125a-5p-002198 | 0.585 |
| hsa-miR-345-002186 | 0.575 |
| hsa-miR-106b-000442 | 0.567 |
| hsa-miR-23b-000400 | 0.566 |
| hsa-miR-224-002099 | 0.543 |
| hsa-miR-200c-002300 | 0.538 |
| hsa-miR-652-002352 | 0.538 |
| hsa-miR-149-002255 | 0.537 |
| hsa-let-7b-002619 | 0.519 |
| hsa-miR-331-000545 | 0.519 |
| hsa-miR-99b-000436 | 0.514 |
| hsa-miR-27a-000408 | 0.511 |
| hsa-miR-324-3p-002161 | 0.499 |
| hsa-miR-222-002276 | 0.482 |
| hsa-miR-342-3p-002260 | 0.479 |
| hsa-miR-155-002623 | 0.478 |
| hsa-miR-20b-001014 | 0.466 |
| hsa-miR-204-000508 | 0.447 |
| hsa-miR-19a-000395 | 0.445 |
| hsa-miR-324-5p-000539 | 0.436 |
| hsa-let-7c-000379 | 0.435 |
| hsa-miR-15b-000390 | 0.424 |
| hsa-miR-146a-000468 | 0.397 |
| hsa-miR-486-001278 | 0.388 |
| hsa-miR-103-000439 | 0.362 |
| hsa-miR-126-002228 | 0.344 |
| hsa-miR-196b-002215 | 0.342 |
| hsa-miR-339-5p-002257 | 0.337 |
| hsa-miR-454-002323 | 0.328 |
| hsa-miR-105-002167 | 0.320 |
| hsa-miR-34a-000426 | 0.300 |
| hsa-miR-485-3p-001277 | 0.274 |
| hsa-miR-9-000583 | 0.249 |
| hsa-miR-330-000544 | 0.221 |
| hsa-miR-181a-000480 | 0.221 |
| hsa-miR-489-002358 | 0.175 |
| hsa-miR-195-000494 | 0.175 |
| hsa-miR-452-002329 | 0.141 |
| hsa-miR-182-002334 | 0.137 |
| hsa-miR-642-001592 | 0.135 |
| mmu-miR-124a-001182 | 0.105 |
| hsa-miR-23a-000399 | 0.072 |
| hsa-miR-142-5p-002248 | 0.045 |
| hsa-miR-874-002268 | 0.045 |
| hsa-miR-597-001551 | 0.023 |

B. miRNA expression of 1889c Dx treatment detected by Quantstudio 12K Flex Pool B (ctrl=1)

| **Target** | **RQ** |
| --- | --- |
| hsa-miR-340#-002259 | 100.937 |
| hsa-miR-638-001582 | 33.636 |
| hsa-miR-149#-002164 | 31.293 |
| hsa-miR-34b-002102 | 29.506 |
| hsa-miR-601-001558 | 24.876 |
| hsa-miR-624-001557 | 23.251 |
| hsa-miR-92a-1#-002137 | 21.345 |
| hsa-miR-378-002243 | 11.757 |
| hsa-miR-320B-002844 | 10.266 |
| hsa-miR-657-001512 | 9.212 |
| hsa-miR-548M-002775 | 8.91 |
| hsa-miR-1249-002868 | 7.983 |
| hsa-miR-144#-002148 | 7.335 |
| hsa-miR-639-001583 | 7.234 |
| hsa-miR-1233-002768 | 6.827 |
| hsa-miR-30d#-002305 | 6.198 |
| hsa-miR-571-001613 | 6.031 |
| hsa-miR-656-001510 | 5.843 |
| hsa-miR-151-5P-002642 | 5.791 |
| hsa-miR-1825-002907 | 5.649 |
| hsa-miR-593-001547 | 5.133 |
| hsa-miR-181a-2#-002317 | 5.126 |
| hsa-miR-550-001544 | 4.777 |
| hsa-miR-151-3p-002254 | 4.374 |
| hsa-miR-1208-002880 | 4.305 |
| hsa-miR-335#-002185 | 3.9 |
| hsa-miR-573-001615 | 3.838 |
| hsa-miR-206-000510 | 3.359 |
| hsa-miR-1180-002847 | 3.148 |
| hsa-miR-1274A-002883 | 3.147 |
| hsa-miR-1285-002822 | 2.997 |
| hsa-miR-767-5p-001993 | 2.933 |
| hsa-miR-1274B-002884 | 2.765 |
| hsa-miR-93#-002139 | 2.38 |
| hsa-miR-664-002897 | 2.214 |
| hsa-miR-939-002182 | 2.138 |
| hsa-miR-106b#-002380 | 2.111 |
| hsa-miR-30a-5p-000417 | 2.036 |
| hsa-miR-30d-000420 | 1.955 |
| hsa-miR-1275-002840 | 1.928 |
| hsa-miR-520c-3p-002400 | 1.838 |
| hsa-miR-720-002895 | 1.745 |
| hsa-miR-18a#-002423 | 1.693 |
| hsa-miR-935-002178 | 1.68 |
| hsa-miR-564-001531 | 1.632 |
| hsa-miR-625#-002432 | 1.631 |
| hsa-miR-1201-002781 | 1.579 |
| hsa-miR-1270-002807 | 1.579 |
| hsa-miR-193b#-002366 | 1.476 |
| hsa-miR-183#-002270 | 1.409 |
| hsa-miR-374b#-002391 | 1.409 |
| hsa-miR-589-001543 | 1.361 |
| hsa-miR-1179-002776 | 1.326 |
| hsa-miR-96#-002140 | 1.276 |
| hsa-miR-30a-3p-000416 | 1.247 |
| hsa-miR-1260-002896 | 1.198 |
| hsa-miR-1303-002792 | 1.185 |
| hsa-miR-30e-3p-000422 | 1.132 |
| hsa-miR-942-002187 | 1.032 |
| hsa-miR-1286-002773 | 0.954 |
| hsa-miR-595-001987 | 0.941 |
| hsa-miR-550-002410 | 0.939 |
| hsa-miR-766-001986 | 0.859 |
| hsa-miR-941-002183 | 0.827 |
| hsa-miR-27a#-002445 | 0.794 |
| hsa-miR-1183-002841 | 0.682 |
| hsa-miR-1225-3P-002766 | 0.679 |
| hsa-miR-1247-002893 | 0.606 |
| hsa-miR-1276-002843 | 0.605 |
| hsa-miR-518f#-002387 | 0.509 |
| hsa-miR-425#-002302 | 0.499 |
| hsa-miR-1254-002818 | 0.399 |
| hsa-miR-629-001562 | 0.299 |
| hsa-miR-25#-002442 | 0.293 |
| hsa-miR-1300-002902 | 0.26 |
| hsa-miR-1269-002789 | 0.228 |
| hsa-miR-92b#-002343 | 0.21 |
| hsa-miR-1291-002838 | 0.188 |
| hsa-miR-520D-3P-002743 | 0.151 |
| hsa-miR-888#-002213 | 0.116 |
| hsa-miR-130b#-002114 | 0.078 |
| hsa-miR-99b#-002196 | 0.039 |

C. miRNA expression of 1889c Dx treatment detected by NanoString nCounter SPRINT Profiler (ctrl=1)

| **Target** | **RQ** |
| --- | --- |
| hsa-miR-551a | 1.896221 |
| hsa-miR-107 | 1.606129 |
| hsa-miR-301a-5p | 1.517176 |
| hsa-miR-200a-3p | 1.514612 |
| hsa-miR-551b-3p | 1.468508 |
| hsa-miR-324-3p | 1.391314 |
| hsa-miR-302d-3p | 1.387764 |
| hsa-miR-598-3p | 1.383155 |
| hsa-miR-363-3p | 1.373447 |
| hsa-miR-1197 | 1.343008 |
| hsa-miR-6721-5p | 1.330568 |
| hsa-miR-2110 | 1.31116 |
| hsa-miR-421 | 1.31116 |
| hsa-miR-607 | 1.295402 |
| hsa-miR-543 | 1.284862 |
| hsa-miR-23a-3p | 1.283406 |
| hsa-miR-4443 | 1.258618 |
| hsa-miR-4755-5p | 1.258564 |
| hsa-miR-495-3p | 1.185658 |
| hsa-miR-378f | 1.171915 |
| hsa-miR-574-5p | 1.165243 |
| hsa-miR-379-5p | 1.155862 |
| hsa-miR-199a-3p+hsa-miR-199b-3p | 1.151149 |
| hsa-miR-1268b | 1.145916 |
| hsa-miR-325 | 1.139838 |
| hsa-miR-378h | 1.137185 |
| hsa-miR-1305 | 1.136597 |
| hsa-miR-183-5p | 1.133076 |
| hsa-miR-548v | 1.120932 |
| hsa-miR-922 | 1.120763 |
| hsa-miR-5196-5p | 1.101436 |
| hsa-miR-585-3p | 1.096011 |
| hsa-miR-548k | 1.075138 |
| hsa-miR-92b-3p | 1.050761 |
| hsa-let-7b-5p | 1.037231 |
| hsa-miR-1180-3p | 1.0314 |
| hsa-miR-1297 | 1.029352 |
| hsa-miR-548y | 1.022539 |
| hsa-miR-1972 | 1.013237 |
| hsa-miR-212-3p | 0.996447 |
| hsa-miR-520d-3p | 0.985113 |
| hsa-miR-1285-5p | 0.981298 |
| hsa-miR-191-5p | 0.98036 |
| hsa-miR-125b-5p | 0.962215 |
| hsa-miR-3074-3p | 0.955606 |
| hsa-miR-532-3p | 0.955606 |
| hsa-miR-203a-5p | 0.954864 |
| hsa-miR-2053 | 0.952763 |
| hsa-miR-511-5p | 0.934068 |
| hsa-miR-656-3p | 0.922563 |
| hsa-miR-378i | 0.896914 |
| hsa-miR-423-3p | 0.894548 |
| hsa-miR-590-5p | 0.894377 |
| hsa-miR-548d-3p | 0.8873 |
| hsa-miR-4536-5p | 0.884682 |
| hsa-miR-548a-5p | 0.879052 |
| hsa-miR-4454+hsa-miR-7975 | 0.877918 |
| hsa-miR-3161 | 0.875802 |
| hsa-miR-584-3p | 0.873837 |
| hsa-miR-522-3p | 0.868282 |
| hsa-miR-320d | 0.86123 |
| hsa-miR-1185-2-3p | 0.857196 |
| hsa-miR-1257 | 0.851244 |
| hsa-miR-4455 | 0.830301 |
| hsa-let-7a-5p | 0.822075 |
| hsa-miR-92a-3p | 0.814132 |
| hsa-miR-197-3p | 0.810124 |
| hsa-miR-3136-5p | 0.808832 |
| hsa-miR-549a | 0.806448 |
| hsa-miR-888-5p | 0.806265 |
| hsa-miR-548ah-5p | 0.798892 |
| hsa-let-7e-5p | 0.786543 |
| hsa-miR-99b-5p | 0.773073 |
| hsa-miR-744-5p | 0.772202 |
| hsa-miR-877-5p | 0.772202 |
| hsa-miR-499a-5p | 0.765591 |
| hsa-miR-3144-3p | 0.747301 |
| hsa-miR-519d-3p | 0.730969 |
| hsa-miR-503-5p | 0.709223 |
| hsa-miR-30e-5p | 0.698224 |
| hsa-miR-298 | 0.689922 |
| hsa-miR-548j-3p | 0.688266 |
| hsa-miR-597-5p | 0.688266 |
| hsa-miR-1290 | 0.683848 |
| hsa-let-7i-5p | 0.656886 |
| hsa-miR-2116-5p | 0.643176 |
| hsa-miR-423-5p | 0.640463 |
| hsa-miR-125a-5p | 0.621699 |
| hsa-miR-548g-3p | 0.613091 |
| hsa-miR-644a | 0.572765 |
| hsa-miR-331-3p | 0.554771 |
| hsa-miR-4531 | 0.553534 |
| hsa-miR-548n | 0.519321 |
| hsa-miR-25-3p | 0.518139 |
| hsa-miR-15b-5p | 0.501381 |
| hsa-miR-1260a | 0.498251 |
| hsa-miR-93-5p | 0.498213 |
| hsa-miR-548aa+hsa-miR-548t-3p | 0.490537 |
| hsa-miR-181a-5p | 0.484005 |
| hsa-miR-1253 | 0.481625 |
| hsa-miR-548q | 0.455658 |
| hsa-miR-122-5p | 0.442079 |
| hsa-miR-627-5p | 0.367072 |
| hsa-miR-1246 | 0.294466 |
| hsa-miR-603 | 0.261783 |
| hsa-miR-494-3p | 0.019722 |
| hsa-miR-579-3p | 0.001881 |
